# A collagen-fibrin patch (Tachosil®) for the prevention of symptomatic lymphoceles after pelvic lymphadenectomy in women with gynecologic malignancies: a randomized clinical trial

**DOI:** 10.1186/1471-2407-14-635

**Published:** 2014-08-30

**Authors:** Christoph Grimm, Stephan Polterauer, Samir Helmy, David Cibula, Michal Zikan, Alexander Reinthaller, Clemens Tempfer

**Affiliations:** Department of General Gynecology and Gynecologic Oncology, Comprehensive Cancer Center - Gynecologic Cancer Unit, Medical University of Vienna, Waehringer Guertel 18-20, 1090 Vienna, Austria; Department of Obstetrics and Gynecology, Gynecologic Oncology Center, Charles University in Prague, Prague, Czech Republic; Department of Obstetrics and Gynecology, Ruhr University Bochum, Bochum, Germany

**Keywords:** Collagen-fibrin patch, Tachosil, Lymphocele, Pelvic lymphadenectomy, Gynecological malignancy

## Abstract

**Background:**

Lymphoceles are a common complication after pelvic lymphadenectomy in women with gynecologic malignancies. Although typically asymptomatic, lymphoceles can superinfect requiring medical or surgical intervention. A single center randomized controlled trial provided first evidence, that a collagen-fibrin patch (Tachosil®) is effective in the prevention of symptomatic lymphoceles after pelvic lymphadenectomy.

**Methods/Design:**

We will perform a multicentre, blinded, randomized, controlled trial comprising 140 women with gynecologic malignancies undergoing pelvic lymphadenectomy. Women will be randomly allocated to Tachosil® application or no application. Primary outcome is efficacy, defined as lymphocele CTCAE 4.03 grade ≥2 within four weeks after surgery. Secondary outcomes are asymptomatic lymphocele verified by ultrasound, medical or surgical intervention. Assuming a two-sided 5% significance level, a power of 80%, and a drop out rate of 10%, a sample size of 68 patients per group was calculated to detect a 66% absolute decrease in symptomatic lymphoceles.

**Discussion:**

We aim to provide further evidence for the efficacy of a collagen-fibrin patch in the prevention of symptomatic lymphoceles in women with gynecological malignancies undergoing pelvic lymphadenectomy.

**Trial registration:**

This study is registered at ClinicalTrials.gov (NCT01470677, protocol ID: TACHO-1). This study is registered at the EudraCT database (EudraCT number: 2011-003115-34).

## Background

Women with gynecologic malignancies such as cervical and endometrial cancer routinely undergo pelvic lymphadenectomy based on tumor characteristics assessed prior to or during surgery. Pelvic lymphadenectomy may be performed by open surgery or laparoscopy [1–4]. The rate of intraoperative complications during and after laparoscopic and open pelvic lymph node dissection is generally low, but has potential long-term consequences such as lower extremity lymph edema, nerve injury, and chronic pain. O’Hanian et al., for example, describe an intraoperative complication rate of 7% in a series of 30 patients with pelvic and paraaortic lymphadenectomy [1]. In a series of 257 women with cervical cancer undergoing laparoscopically assisted radical trachelectomy and pelvic lymphadenectomy, Marchiole et al. describe 11 (4%) intraoperative complications with 5 (2%) of these attributable to lymphadenectomy, i.e. vascular and ureteral injury [2]. Xu et al. performed laparoscopic pelvic lymphadenectomies in 313 patients with cervical cancer, 143 of which also underwent paraaortic lymphadenectomy and report 7 (2%) cases of intraoperative complications possibly related to lymphadenectomy [3]. Postoperative complications during and after pelvic lymphadenectomy include local abscess, bleeding, lymphocele, and chronic lymphedema of the lower extremities, which has an incidence of 1 to 2% [2, 3]. The rate of complications does not seem to differ significantly in women undergoing open or laparoscopic lymphadenectomy based on the available literature [1–6].

In the present trial, we will focus on pelvic lymphoceles, one of the most common complications of pelvic lymphadenectomy. Simonato et al. described a rate of 19/30 (63%) of sonographically detected lymphoceles in men undergoing pelvic extraperitoneal lymphadenectomy for prostate cancer [4]. In this trial, 4/19 men with lymphoceles were symptomatic and required medical interventions. In women with cervical cancer, asymptomatic lymphoceles detected by ultrasound have been noted in up to 11% of women after pelvic lymphadenectomy [1, 3, 5]. In 2% of women, clinical symptoms will require a therapeutic intervention [6].

Tachosil® is a fibrin-collagen coated patch and has been licensed in 2004 in Europe for surgical use in humans to support surgical hemostatic interventions. The efficacy and safety of Tachosil® has been demonstrated in liver resection, pulmonary lobectomy, and kidney tumor resection trials [7–9]. In men, but not in women, it has been demonstrated that the application of a collagen-fibrin patch to the lymphadenectomy surgery site may prevent a significant proportion of lymphoceles. In a randomized trial, Simonato et al. found that the pelvic application of two Tachosil® patches to the obturator fossa and the femoral canal was sufficient to significantly reduce the rate of sonographically detected lymphoceles within 4 weeks after surgery from 19/30 to 5/30 cases (p = 0.001) as well as the mean drainage volume from 190 to 64 ml. Percutaneous puncture of a symptomatic lymphocele was necessary in 1/30 individuals in the intervention group compared to 4/30 individuals in the control group [4]. In women with gynecological malignancies a single center randomized controlled trial has found, that Tachosil® seems effective to reduce the rate of lymphoceles after pelvic lymphadenectomy [5]. 7/30 (23.3%) women in the treatment group compared to 9/28 (57.7%) women in the control group developed asymptomatic lymphoceles (p < 0.05) [5]. No significant differences between the two groups were observed in the development of symptomatic lymphoceles or the rate of interventions [5]. This may be attributable to the small sample size of this study. As symptomatic lymphoceles are more relevant for the patient, it seems clinically more important to evaluate the impact of Tachosil® on the rate of symptomatic lymphoceles after pelvic lymphadenectomy.

Symptomatic lymphoceles are defined by the CTCAE 4.03 grading system as lymphoceles grade ≥2. This includes all lymphoceles needing medical intervention. Thus this definition comprises, lymphoceles with the presence of localized pelvic pain, pelvic abscess, fever, and/or leg edema in the presence of a sonographically verified pelvic lymphocele.

In summary, the data available in the literature demonstrate that pelvic lymphoceles occur in 11 to 63% of individuals undergoing pelvic lymphadenectomy. Symptomatic lymphoceles seem to occur in about 32% of patients undergoing pelvic lymphadenectomy. Intraoperative application of a collagen-fibrin patch may reduce the number of lymphoceles, mean drainage volume, and the necessity of medical interventions such as percutaneous puncture.

Therefore, we intend to perform a multi center randomized clinical trial assessing the efficacy of a collagen-fibrin patch for preventing symptomatic lymphoceles in women undergoing pelvic lymphadenectomy for gynecologic malignancies, ie cervical and endometrial cancer. We hypothesize that, based on the data of Simonato et al. and Tinelli et al., the application of a collagen-fibrin patch (Tachosil®) will reduce the number of symptomatic pelvic lymphoceles by at least 66%.

## Methods/Design

### Aim of the study

Primary outcome variable:

2.1.1. To evaluate the incidence of symptomatic pelvic lymphoceles defined by CTCAE 4.03 grade ≥2 within 4 weeks after surgery in women undergoing open or laparoscopic pelvic lymphadenectomy for cervical and endometrial cancer with and without the application of Tachosil® during surgery.

Secondary outcome variables:

2.1.2 To evaluate the incidence of sonographically detected pelvic lymphoceles of at least 2 cm in the largest diameter 4 weeks after surgery in women undergoing open or laparoscopic pelvic lymphadenectomy for cervical and endometrial cancer with and without the application of Tachosil® during surgery.

2.1.3. To evaluate the rate and type of medical interventions for clinically symptomatic pelvic lymphoceles such as analgesics and/or lymphocele puncture and drainage.

2.1.4. To evaluate the rate of total and symptomatic lymphoceles between the three centers.

2.1.5. To evaluate the rate of total and symptomatic lymphoceles between cervical and endometrial cancer patients.

2.1.6. To evaluate the rate of total and symptomatic lymphoceles between the surgical device used for pelvic lymphadenectomy.

2.1.7. To evaluate the rate of total and symptomatic lymphoceles between patients with and without lymph node metastases.

2.1.8. To evaluate the risk for total and symptomatic lymphoceles depending on the total number of pelvic lymph nodes removed.

2.1.9. To evaluate the rate of total and symptomatic lymphoceles between patients with open and laparoscopic pelvic lymphadenectomy.

### Study hypothesis

We hypothesize that the intraoperative application of two collagen-fibrin patches (Tachosil®) to the obturator fossa and the femoral canal will reduce the number of symptomatic pelvic lymphoceles by at least 66% (primary study end point).

### Study design

Prospective randomized clinical intervention trial of 140 women undergoing open or laparoscopic pelvic lymphadenectomy for cervical or endometrial cancer. Randomization will be by a computerized randomization list. Women will be centrally randomized by the principal investigator (CT). Allocation will be communicated by telephone after informed consent has been obtained and after lymphadenectomy has been completed. This is a single-blinded study, ie patients, but not surgeons, will be blinded to the treatment allocation. Outcome assessment will not be performed by the surgeon, who has performed the lymphadenectomy. Outcome assessors will be blinded to the treatment allocation.

### Inclusion criteria

- Women undergoing open or laparoscopic surgery for cervical or endometrial cancer.
- Age between 18 and 70 yrs.
- Informed consent.

### Exclusion criteria

- Women with previously diagnosed lymph edema.
- Known disease of the lymphatic system.
- Immunocompromised women such as those with an immunosuppressive medication or a known disease of the immune system.

### Treatment

**Figure 1:**
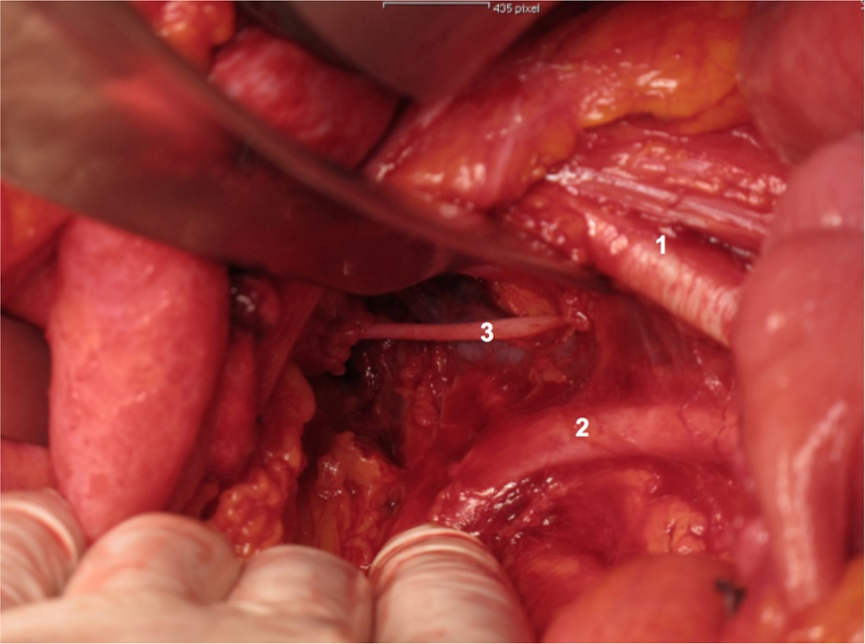
**Right pelvic side wall after systematic pelvic lymphadenectomy.** 1 = Right external iliac artery. 2 = Right internal iliac artery, 3 = Right obturator nerve.

**Figure 2:**
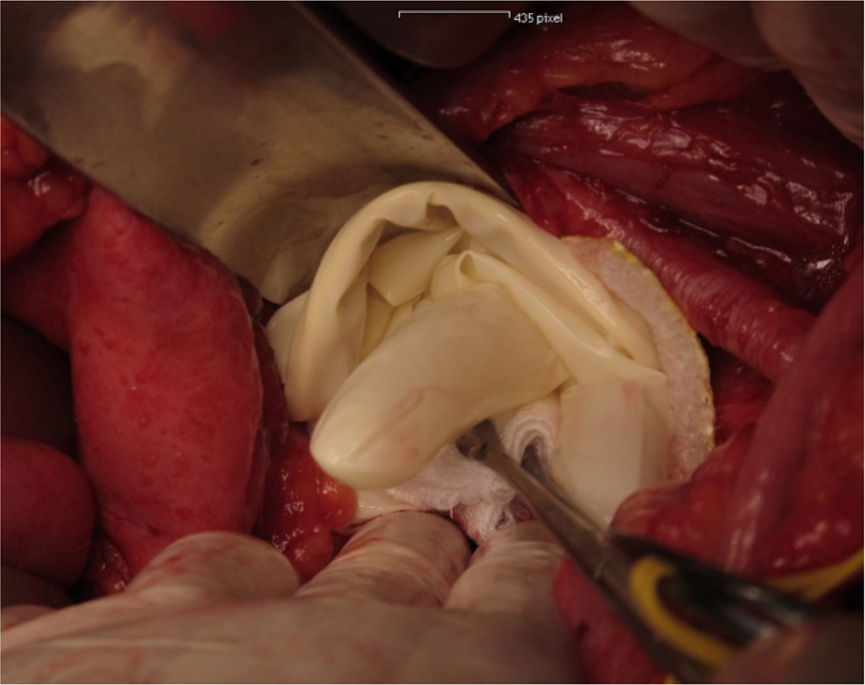
**Tachosil® is applied with a wet glove and sponge and compression for 30–60 seconds.** Thereafter glove and sponge are removed.

**Figure 3:**
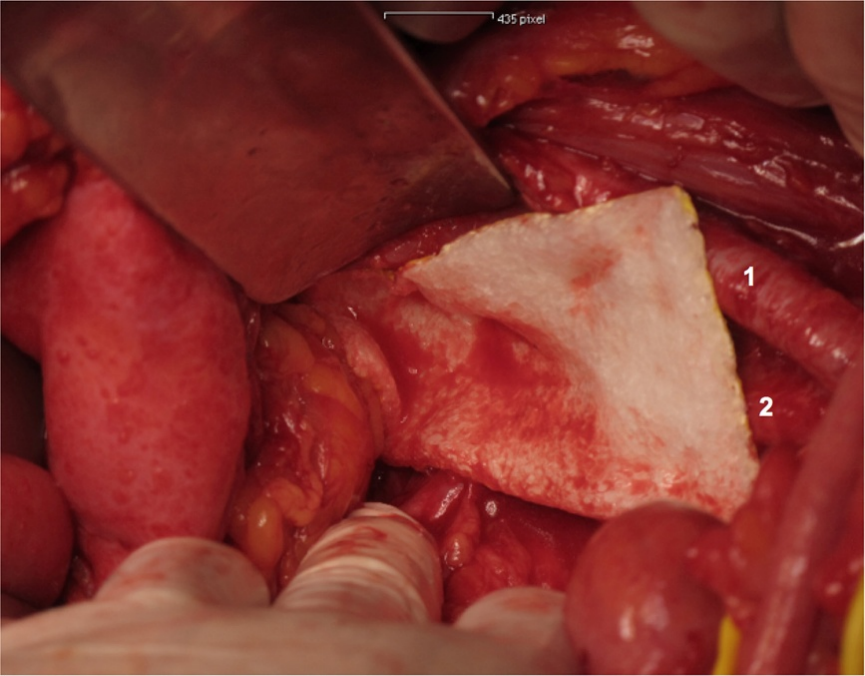
**Placement of the first Tachosil patch in the right obturator fossa.** 1 = Right external iliac artery. 2 = Right internal iliac artery.

All women will undergo open or laparoscopic surgery. Within this procedure, as deemed appropriate by the surgeon, women will undergo pelvic lymphadenectomy. The procedure will be performed as follows:The peritoneum will be incised parallel to the iliac vessels. Then, the iliac vessels will be screened for the presence of bulky lymph nodes. If a lymph node debulking is performed, no patch will be applied. In women who undergo routine pelvic lymphadenectomy, lymph node tissue will be removed from the external iliac vessels, the obturator fossa, the interiliac region, and the common iliac region after identification and appropriate preparation of surgical landmarks, ie iliac vessels, femoral canal, chorda, and obturator nerve. At the end of the procedure, hemostasis will be checked. A Tachosil® patch of 4.8x4.8 cm will be attached to the obturator fossa and a Tachosil® patch of 4.8x4.8 cm will be attached to the femoral canal of each side of surgery in the intervention group (Figures 1, 2 and 3). In the control group, no Tachosil® patch will be used. No specific drainage of the retroperitoneum will be performed. The study team members Clemens Tempfer, David Cibula, and Alexander Reinthaller, experienced in open and laparoscopic pelvic lymph node dissection, will perform all surgical procedures. In order to ensure adequate application of the Tachosil® patch by laparoscopy, all surgeons will perform at least two laparoscopic training operations during which they roll the Tachosil® patch around a laparoscopic instrument, move it through a 10 mm trocar into the abdomen, and flatten it out.

Patients have to agree to participate in the study and sign informed consent at least one day before surgery. If patients are eligible and agreed to participate, they are included in consecutive order. Patients receive an envelope according to their inclusion number in which allocation is documented. This envelope accompanies the patient into the operating room. A nurse opens the envelope not before pelvic lymphadenectomy and hemostasis have been completely finished. According to the treatment group the patient is allocated, the surgeon now has to apply either two Tachosil® patches in the intervention group or no Tachosil® in the control group. Therefore, the surgeon cannot influence the extent of the lymphadenectomy, as he is not aware of the patient’s allocation until the end of the lymphadenectomy.

### Study population

The study population consists of 140 women undergoing pelvic lymphadenectomy for cervical and endometrial cancer in one of the three study sites, ie the Departments of Obstetrics and Gynecology at the Ruhr University Bochum, the Gynecologic Oncology Center at the Charles University in Prague, and the Medical University of Vienna fulfilling the inclusion/exclusion criteria requirements.

### Outcome variables

2.8.1. The incidence of symptomatic pelvic lymphoceles CTCAE 4.03 grade ≥2 within 4 weeks after surgery in women undergoing open or laparoscopic pelvic lymphadenectomy for cervical or endometrial cancer (primary outcome variable).

2.8.2. The incidence of sonographically detected pelvic lymphoceles of at least 2 cm in the largest diameter 4 weeks after surgery in women undergoing open or laparoscopic pelvic lymphadenectomy for cervical and endometrial cancer with and without the application of Tachosil® during surgery (secondary outcome variable).

2.8.3. The rate and type of medical interventions for clinically symptomatic pelvic lymphoceles such as analgesics and/or lymphocele puncture and drainage (secondary outcome variable).

2.8.4. The length of hospital stay (secondary outcome variable).

2.8.5. The rate of predefined complications, ie abscess in the pelvis, fever >38°C on at least two occasions >24 hrs apart, leg swelling, local pelvic bleeding complications necessitating a surgical intervention (secondary outcome variable).

### Statistical analysis

A power calculation demonstrated that, with a sample size of 70 per group, a two-arm study has a power of 80% to detect a 66% absolute difference in treatment efficacy at a significance level of 0.05 regarding the primary outcome parameter, ie symptomatic lymphoceles CTCAE 4.03 grade ≥2. This calculation was based on published data by Tinelli et al. observing a rate of 32% of symptomatic lymphoceles in the placebo group and 10% in the Tachosil® group [5]. Assuming a 10% drop out-rate, 140 women will be randomized. The chi-square test will used for comparisons of frequencies and cross-tabulations. One Way ANOVA on ranks will be used on means. Descriptive statistics (means, standard deviations, and ranges) will be used for demographic data. Bonferroni’s correction will be used for multiple comparisons of secondary outcomes.

### Risk benefit assessment

The expected rate of intraoperative and postoperative complications following the study-based additional intervention, i.e. application of two patches of Tachosil®, will be minimal based on published studies [4–9]. For example, Simonato et al. and Tinelli et al. reported no complication associated with the use of Tachosil® patches [4, 5]. However, local infectious complications, pain, and abscess cannot be ruled out. A patient insurance for all participating women will be contracted covering all eventual harm caused by study participation. Also, the duration of the surgical intervention will not be increased due to the study intervention in a relevant manner [4, 5].

Women recruited for this study may eventually benefit from participating since a previous randomized trial in men undergoing pelvic lymphadenectomy has resulted in a significant benefit of Tachosil® regarding occurrence of lymphoceles and lymphocele drainage volume. Also, the number of medical interventions such as percutaneous punctures may be reduced in the intervention group.

The putative findings of our study could help to modify one step of the surgical technique of pelvic lymphadenectomy, one of the most common gynecologic oncology surgical procedures.

The present study has been approved the Ethics Committee of the Medical University of Vienna, Austria by September 9^th^, 2011 (EC no.: 740/2011), of the Charles University in Prague, Czech Republic by March 15^th^, 2012 (EC no.: cj459/12), and of the Marienhospital Bochum, Germany by August 24^th^, 2012 (EC no.: 4299–12 FF).

### Follow-up

All women will undergo a gynecologic examination and a transvaginal and transabdominal ultrasound examination at the time of discharge of the hospital, performed by a physician experienced in transvaginal and transabdominal ultrasound examinations, who has not participated in the original surgical procedure and is blinded to the treatment allocation. All women will be scheduled for a follow-up visit 4 weeks after surgery including a gynecologic examination and a transvaginal and transabdominal ultrasound examination, performed by a physician experienced in transvaginal and transabdominal ultrasound examinations, who has not participated in the original surgical procedure and is blinded to the treatment allocation.

### Data management and safety

Patients will be pseudo-anonymized for protection of data privacy. At the time of inclusion, a code based on initial of given name and surname and their date of birth will be assigned to every patient. The code will only be used for data entry in the database, as the patient’s full name is not documented in the same database, where clinical information is collected and stored. During the clinical routine and follow-up visits patient information is documented on study documents, where both, patient’s full name and patient’s code, have to be documented. Only the principal investigators (CT, DC, and AR) will have access to study data.

## Discussion

The present randomized, controlled trial aims to evaluate the efficacy of Tachosil® in the prevention of symptomatic lymphoceles after pelvic lymphadenectomy in gynaecological malignancies. This is a clinical relevant topic as along with lymphedema, symptomatic lymphoceles are the major complication after pelvic lymphadenectomy. Recently, a study in patients with endometrial cancer described a prevalence of 23% for lymphedema after pelvic lymphadenectomy [10]. Due to the importance of this topic a prospective trial led by the Gynecologic Oncology Group has been released (GOG 244, GOG LEG study, The Lymphedema and Gynecologic Cancer Study).

In contrast to lymphedema, which is rather a chronic problem after pelvic lymphadenectomy, lymphoceles are more of an acute complication. The prevalence of lymphoceles after pelvic lymphadenectomy provided in the literature varies quite substantially between 11-63% [4, 5]. Symptomatic lymphoceles seem to occur in about 32% of patients undergoing pelvic lymphadenectomy [5]. This variation might be caused by numerous factors: the time interval between surgery and follow-up visit for lymphoceles as most of the small lymphoceles will spontaneously regress over time, rather small study populations in the majority of trials, the extent of lymphadenectomy, the use of new energy sources [5, 11]. Another interesting factor possibly influencing the rate of lymphoceles after pelvic lymphadenectomy might be the surgical approach. One study revealed a postoperative lymphocele rate of 15.4% and 1.4% in women after laparoscopic and after open pelvic lymphadenectomy, respectively [12]. Of note, this study retrospectively compared 138 women, who underwent systematic laparoscopic pelvic lymphadenectomy, to 123 historical control subjects, who underwent systematic pelvic lymphadenectomy via an open approach. Due to the design, this study has to be interpreted with caution [12]. In the present study, we aim to prospectively describe the lymphocele rates following open compared to laparoscopic systematic pelvic lymphadenectomy in women with gynecological malignancy.

The value of Tachosil® in the prevention of lymphoceles after pelvic lymphadenectomy in women has already been studied in two clinical trials [5, 13]. In a retrospective matched case–control study, a cohort of 26 patients receiving Tachosil® after laparoscopic pelvic lymphadenectomy was compared to a historical cohort of 29 women without Tachosil® after laparoscopic pelvic lymphadenectomy. The study described a significant difference in the prevalence of total lymphoceles (19.2% of patients in the Tachosil® group vs. 51.7% in the historical control group) and a non-significant difference of symptomatic lymphoceles (7.6% of patients in the Tachosil® group vs. 17.2% in the historical control group). Of note, this was not a prospective trial with a limited number of patients [13]. The second study was a prospective randomized controlled trial comprising 58 women with endometrial cancer after undergoing pelvic lymphadenectomy with or without Tachosil® [5]. This trial observed, that Tachosil® seems effective to reduce the rate of lymphoceles after pelvic lymphadenectomy [5]. 7/30 (23.3%) women in the treatment group compared to 9/28 (57.7%) women in the control group developed asymptomatic lymphoceles (p < 0.05) [5]. No significant differences between the two groups were observed in the development of symptomatic lymphoceles or the rate of interventions [5]. This may be attributable to the small sample size of this study.

As symptomatic lymphoceles are more relevant for the patient, it seems clinically more important to evaluate the impact of Tachosil® on the rate of symptomatic lymphoceles after pelvic lymphadenectomy. Therefore, we intend to perform a multi center randomized clinical trial assessing the efficacy of a collagen-fibrin patch for preventing symptomatic lymphoceles in women undergoing pelvic lymphadenectomy for gynecologic malignancies, ie cervical and endometrial cancer. We hypothesize that based on the data of *Simonato et al.* and *Tinelli et al.*, the application of a collagen-fibrin patch (Tachosil®) will reduce the number of symptomatic pelvic lymphoceles by at least 66%.

## Conclusion

Despite being one of the major complications of lymphadenectomy, little is known about the prevention of lymphoceles. One prospective controlled trial comprising 58 women already evaluated the preventative effect of Tachosil® in the prevalence of lymphoceles (5). As this trial was not adequately powered to detect differences between symptomatic lymphoceles, we designed the present open, randomized, controlled trial.

## Abbreviations

MD: Medical doctor
CTCAE: Common terminology criteria for adverse events
C: Celsius
Hrs: hours.
